# Potent and Synergistic Extract Combinations from *Terminalia Catappa, Terminalia Mantaly* and *Monodora tenuifolia* Against Pathogenic Yeasts

**DOI:** 10.3390/medicines2030220

**Published:** 2015-08-26

**Authors:** Thierry Kammalac Ngouana, Cedric Derick Jiatsa Mbouna, Rufin Marie Toghueo Kuipou, Marthe Aimée Tchuente Tchuenmogne, Elisabeth Menkem Zeuko’o, Vincent Ngouana, Michèle Mallié, Sebastien Bertout, Fabrice Fekam Boyom

**Affiliations:** 1 Antimicrobial Agents Unit, Laboratory for Phytobiochemistry and Medicinal Plants Studies, Department of Biochemistry, Faculty of Science, University of Yaoundé I, P.O. Box 812, Messa-Yaoundé, Cameroon; E-Mails: ngouanathi@yahoo.com (T.K.N.); cedrickjiatsa@yahoo.com (C.D.J.M.); toghueo.rufin@yahoo.fr (R.M.T.K.); zeukoo@yahoo.com (E.M.Z.); vngouana@yahoo.fr (V.N.); 2 Clinical Biology Laboratory, Yaoundé Central Hospital, P.O. Box 87, Messa-Yaoundé, Cameroon; 3 IRD UMI 233 TransVIHMI—UM INSERM U1175 TransVIHMI Laboratoire de Parasitologie et Mycologie Médicale, UFR Pharmacie, Université de Montpellier 1, 15, Avenue Charles Flahault—BP 14491 34093 Montpellier, France; E-Mails: michele.mallie@univ-montp1.fr (M.M.); sebastien.bertout@univ-montp1.fr (S.B.); 4 Laboratory of Natural Products and Organic Synthesis, Department of Organic Chemistry, Faculty of Science, University of Yaoundé 1, P.O. Box 812, Messa-Yaoundé, Cameroon; E-Mail: tch_aimee@yahoo.fr

**Keywords:** *Terminalia catappa*, *Terminalia mantaly*, *Monodora tenuifolia*, antifungal activity, bio-guided fractionation, combinations

## Abstract

Mycoses caused by *Candida* and *Cryptococcus* species, associated with the advent of antifungal drug resistance have emerged as major health problems. Improved control measures and innovative therapies are needed. This paper describes results from the screening of bio-guided fractionated extracts alone and combinations of *Terminalia catappa, Terminalia mantaly* and *Monodora tenuifolia* harvested in Cameroon. Crude ethanolic, hydro-ethanolic and aqueous extracts and bio-guided fractions were screened for antifungal activity against isolates of *C. albicans*, *C. glabrata*, *C. parapsilosis* and *Cr. neoformans* and the reference strain *C. albicans* NR-29450. Minimal inhibitory concentrations (MIC) were determined using a broth micro dilution method according to the Clinical & Laboratory Standards Institute (CLSI). Time kill kinetics of extracts alone and in combination were also evaluated. Extracts from *T. mantaly* stem bark were the most active with the best MIC values ranging from 0.04 mg/mL to 0.16 mg/mL. Synergistic interactions were observed with combinations of sub-fractions from *M. tenuifolia*, *T. mantaly* and *T. catappa.* Combination of sub-fractions from *M. tenuifolia* and *T. mantaly* (C36/C12) showed synergistic interaction and fungicidal effect against four out of five tested yeasts. These results support further investigation of medicinal plant extracts alone and in combination as starting points for the development of alternative antifungal therapy.

## 1. Introduction

The knowledge of the epidemiology and pathogenesis of yeasts affecting humans has increased in the last three decades [1,2,3,4]. Many conditions have promoted the increase of opportunistic fungal infections in humans, especially candidiasis and cryptococcosis [1,3,4,5,6,7,8,9,10,11,12].

Candidiasis is the most common fungal infection in both immunocompromised and immunocompetent persons [10,13,14,15,16,17]. Invasive candidiasis is responsible for the high mortality among intensive care patients with a frequency of 40%–75% [3,9,15,18]. *C. albicans* is the most frequent yeast involved in these infections, although other species are also frequently isolated such as *C. parapsilosis*, *C. glabrata*, *C. tropicalis*, *C. krusei, C. lusitaniae*, and *C. dubliniensis.* Furthermore, some other pathogenic yeasts are emerging, including *C. famata*, *C. guilliermondii*, *C. kefyr* and more recently *C. africana* [9,19]. Moreover, Cryptococcal meningitis caused by *Cr. neoformans* is the most dangerous systemic mycosis associated with AIDS [20,21,22].

Fungal infections are treated using different families of drugs such as polyens, azoles, pyrimidine analogues, echinocandins and allylamines [2,9,23]. However, these drugs have some limitations such as high toxicity and low bioavailability (polyens) and reduced spectrum of action (echinocandins) [9,23]. Resistance has already been described for all these antifungal drugs and is more often associated with therapeutic failure [23,24,25]. These limitations in treatments associated with the increased knowledge of the epidemiology and pathogenicity of yeasts underline the urgent need for new antifungal agents with improved potency and innovative modes of action that could be further developed as antifungal treatments.

Natural products have proven efficacy in the treatment of a wide range of ailments. Ethnobotanically selected plants such as *T. mantaly* (Combretaceae)*, T. catappa* (Combretaceae) and *M. tenuifolia* (Annonaceae) are frequently used in traditional medicine for the treatment of various infectious diseases [26,27]. This paper describes the antifungal activity of their extracts, fractions and sub-fractions against *Candida* spp and *Cr. neoformans* isolates*.*

## 2. Experimental Section

### 2.1. Collection of Plant Materials

Organs from *T. catappa*, *T. mantaly* (Combretaceae) and *M. tenuifolia* (Annonaceae) were collected in the Yaoundé area and identified at the Cameroon National Herbarium where voucher specimens are kept under registration numbers 51244/HNC (*Terminalia catappa* Linné), 64212/HNC (*Terminalia mantaly* H. Perrier) and 30549/HNC (*Monodora tenuifolia* Benth). Leaves, twigs, fruits, fruits pericarp, pulp, seeds, seeds pericarp, branches and stem bark were collected and separately dried at room temperature and ground using a blender.

### 2.2. Yeasts Isolates and Reference Strain

Yeast isolates were provided by the laboratory of clinical biology, Yaoundé Central Hospital (Yaoundé, Cameroon), and consisted of clinical isolates of *C. albicans*, *C. glabrata*, *C. parapsilosis* and *Cr. neoformans. C. albicans* NR-29450 was obtained from BeiResources (Manassas, VA, USA) and used as positive control. These yeasts were maintained at room temperature and cultured at 37 °C for 24 h on Sabouraud Dextrose Agar (Oxoid) slants prior to use.

### 2.3. Plant Extraction and Preliminary Screening of Antifungal Activity

The crude extracts were prepared by maceration of individual plant material in each solvent; ethanol (95%), distilled water and ethanol-water mixture (70:30, *v/v*), for 48 h. Organic solvents were subsequently evaporated from filtrates using a rotary evaporator (Büchi 011, Flawil Switzerland). Water filtrates were dried using a desiccator at 60 °C (Memmert UN30, Schwabach, Germany). Each plant powder was extracted separately three times using the same amount of solvent, and the dried extracts were pooled and weighed. The yields of extraction were calculated in percentage relative to the weight of the starting plant material.

For the preliminary antifungal screening, the crude extracts were dissolved in 10% dimethylsulfoxide (DMSO) and tested using the agar dilution method, according to the CLSI M44-A2 protocol [28,29] with some modifications. Briefly, sterile Mueller Hinton agar medium (Oxoid) supplemented with 2% glucose, was maintained at 45 °C and the extract added to obtain a final concentration of 40 mg/mL. The mixture was then transferred in a Petri dish and let to solidify at room temperature. Plates were inoculated with 2.0 × 10^4^ yeast cells/mL in sterile normal saline solution and incubated at 37 °C for 48 h. Growth control consisted of microorganisms cultured on the Mueller Hinton agar medium without plant extracts. Fluconazole (Sigma-Aldrich, Heidenheim, Germany) was used as positive control at 10 µg/mL. Experiments were run simultaneously and each test performed in duplicate. At incubation time, yeast colonies were enumerated and inhibition percentages (IP) were calculated using the formula:

IP (%) = (colonies in growth control − colonies in test dish) × 100/colonies in growth control

Crude extracts that showed an inhibition percentage of ≥75% against at least one of the tested yeasts were selected for the determination of minimal inhibitory concentration (MIC), defined as the lowest concentration at which there was no visible growth of the yeasts.

MIC values of the selected extracts were determined according to the CLSI M27-A3 protocol [30] with little modifications. Briefly, a serial two-fold dilution of each crude extract using Mueller Hinton broth medium (Oxoid) supplemented with 2% glucose was done in 96-wells microtiter plates starting with 40 mg/mL up to 0.04 mg/mL. One hundred microliters of fungal inocula at the final concentrations of 0.5–2.5 × 10^3^ CFU/mL were then added into each well of the plate to achieve a final volume of 200 µL. Plates were incubated at 37 °C for 48 h. Fluconazole was tested as positive control at the highest concentration of 64 µg/mL. MICs were determined based on turbidity in plate wells through macroscopic observation. Crude extracts that showed MIC ≤ 1.25 mg/mL against at least half of the tested yeasts were selected for progression to the bio-guided fractionation.

### 2.4. Bio-Guided Fractionation of Selected Crude Extracts

Extract fractionation consisted in partitioning 10 g of each selected extract in 300 mL mixture of methylene chloride/water (*v/v*). After exhaustion of the methylene chloride phase, fractions were concentrated and dried. Change in MICs of the dried fractions was determined as described above and promising fractions were selected for progression. Five grams of each selected fraction was further partitioned into 150 mL mixture of hexane/methanol (*v/v*) and the afforded sub-fractions tested for activity.

### 2.5. Antifungal Activity of Combined Sub-Fractions

Seven sub-fractions from the three plants were combined to give 11 interspecies combinations, with no combination between extracts from the same plant. Checkerboard tests were used to determine fractional inhibitory concentration indexes (FICIs) of combinations of sub-fractions against each test microorganism. The checkerboard broth microdilution method based on CLSI recommendations [31] consisted of diluting sub-fractions in the two directions of a 96-wells microplate. Mixed concentrations in wells ranged from 1/8 × MIC to 8 × MIC and 1/128 × MIC to 8 × MIC for the two sub-fractions, respectively. The fungal inoculum was added to give an ultimate concentration of 0.5–2.5 × 10^3^ CFU/mL in a final volume of 100 µL. Plates were therefore incubated at 37 °C for 48 h and each test performed in duplicate.

Changes in the Fractional Inhibitory Concentration Indexes (FICIs) were calculated using the following formula, and the type of interaction was determined according to previously described criteria [31,32].

FICI_(X1/X2)_ = MIC_X1_ in combination*/*MIC_X1_ alone + MIC_X2_ in combination*/*MIC_X2_ alone [33].

Based on these criteria, a combination was considered to be synergistic when the FICI was ≤ 0.5, additive when it was > 0.5 to ≤ 1, indifferent when it was > 1.0 to ≤ 4.0. Antagonism was obtained with FICI > 4.0.

### 2.6. Time Kill Kinetic Assay of the Most Active Combinations

The time kill kinetic assay of the selected combinations was assessed using the method described by Klepser *et al.* [34] with some modifications. The assays were performed in duplicate in 1.5 mL conical tubes at final concentrations of 4xMIC for each sub-fraction in the Mueller Hinton broth. For each combination, three tubes were used, two containing sub-fractions alone and one containing the combination of sub-fractions. The final volume in each tube was 1 mL, containing a fungal inoculum of 1–2.10^5^ cells/mL. After incubation under orbital shaking (IKA-Vibrax-VXR, Radnor, PA, USA) (32× *g*) at 37 °C, and at time intervals of 0, 2, 4, 6, 8, 12, 16 and 24 h, a 50 μL aliquot was collected from each culture to inoculate 950 μL of sterile broth medium and incubated at 37 °C for 24 h to determine the number of cells/mL using a Malassez haemocytometer (Thermo Fisher Scientific, Darmstadt, Germany). Time kill kinetic curves were plotted as log10 (number of viable cells) *versus* time. The following criteria were used to interpret the interactions: Synergy was obtained when the fungicidal effect led to ≥2log10 decrease in cells/mL for the combination compared to the most active sub-fraction; additivity was defined as <2log10 decrease in cells/mL for the combination compared to the most active sub-fraction; indifference as <2log10 increase in cells/mL for the combination compared to the least active sub-fraction; and antagonism as ≥2log10 increase in cells/mL for the combination compared to the least active sub-fraction [9,34].

### 2.7. Phytochemical Screening of Selected Crude Extracts and Sub-Fractions

A qualitative phytochemical analysis of promising extracts and sub-fractions was carried out to identify groups of secondary metabolites known as having antifungal activity, including flavonoids, tannins, anthraquinones, alkaloids, steroids, glucosides, saponins, triterpenes, anthocyanins and phenols and using the previously described protocols [35,36,37,38].

## 3. Results

### 3.1. Plant Extraction and Preliminary Screening of Antifungal Activity

Thirty-four crude extracts were obtained with yields ranging from 1.7%–36.7% relative to the weights of the starting plant materials. Results from the preliminary screening of antifungal activity enabled the selection of 23 extracts based on their inhibitory percentages against clinical isolates and the reference *Candida albicans* NR-29450 (≥75% against at least one of the tested yeasts). Change in MIC values in liquid medium ranging from 0.04 mg/mL to 40.0 mg/mL was observed as described in Table 1. Based on our selection criteria (MIC ≤ 1.25 mg/mL against at least half of tested yeasts), 15 crude extracts were selected and progressed to the bio-guided fractionation, including extracts A1 to A15.

**Table 1:** Minimal inhibitory concentrations (MIC) of crude extracts, fractions and sub-fractions.

**Codes**	**Crude Extracts**	**^a^ MIC (mg/mL)**					
		**Reference Strain**		**Isolates**			
		***C. albicans* NR-29450**		***C. albicans***	***C. glabrata***	***C. parapsilosis***	***Cr. neoformans***
A1	Te C L H_2_O	0.31		2.50	0.63	2.50	0.63
A2	Te C L H_2_O/EthOH	0.08		0.31	0.08	0.31	0.16
A3	Te C L EthOH	0.31		0.16	0.08	0.31	0.31
A4	Te C Sb H_2_O	0.16		0.63	0.31	0.31	0.63
A5	Te C Sb H_2_O/EthOH	0.08		2.50	0.63	0.31	0.63
A6	Te C Sb EthOH	0.16		0.16	0.16	0.16	0.16
A7	Te M L H_2_O	0.31		2.50	0.31	0.31	0.16
A8	Te M L H_2_O/EthOH	0.04		0.04	0.04	0.08	0.08
A9	Te M L EthOH	0.04		0.08	0.08	0.16	0.16
A10	Te M Sb H_2_O	0.08		0.08	0.08	0.08	0.08
A11	Te M Sb H_2_O/EthOH	0.08		0.31	0.16	0.16	0.08
A12	Te M Sb EthOH	0.08		0.16	0.16	0.08	0.04
A13	Mo T L H_2_O	10.00		1.25	0.63	10.00	1.25
A14	Mo T L H_2_O/EthOH	5.00		0.63	0.63	1.25	1.25
A15	Mo T L EthOH	2.50		5.00	1.25	1.25	0.08
A16	Mo T Tw H_2_O	20.00		10.00	5.00	5.00	2.50
A17	Mo T Br H_2_O	10.00		5.00	5.00	2.50	5.00
A18	Mo T Pu H_2_O	2.50		10.00	5.00	5.00	10.00
A19	Mo T Pu H_2_O/EthOH	5.00		5.00	5.00	10.00	2.50
A20	Mo T Pu EthOH	5.00		2.50	5.00	10.00	2.50
A21	Mo T Se H_2_O/EthOH	40.00		10.00	10.00	40.00	40.00
A22	Mo T FrPe EthOH	10.00		5.00	5.00	10.00	2.50
A23	Mo T PSe EthOH	20.00		10.00	20.00	40.00	20.00
**Codes**	**Fractions**	**^a^ MIC (mg/mL)**					
		**Reference Strain**		**Isolates**			
		***C. albicans* NR-29450**		***C. albicans***	***C. glabrata***	***C. parapsilosis***	***Cr. neoformans***
B1	Te C L H_2_O	Fr H_2_O	1.25	1.25	5	0.63	0.16
B2		Fr CH_2_Cl_2_	0.63	1.25	5	0.16	0.16
B3	Te C L H_2_O/EthOH	Fr H_2_O	0.31	2.5	0.63	0.31	0.31
B4		Fr CH_2_Cl_2_	0.31	5	0.63	0.31	0.31
B5	Te C L EthOH	Fr H_2_O	0.63	>5.000	0.63	0.31	0.16
B6		Fr CH_2_Cl_2_	0.63	5	0.63	0.63	0.31
B7	Te C Sb H_2_O	Fr H_2_O	1.25	2.5	2.5	0.63	0.31
B8		Fr CH_2_Cl_2_	1.25	5	5	2.5	0.63
B9	Te C Sb H_2_O/EthOH	Fr H_2_O	0.31	5	2.5	2.5	0.63
B10		Fr CH_2_Cl_2_	0.16	0.63	1.25	1.25	0.63
B11	Te C Sb EthOH	Fr H_2_O	0.16	0.63	2.5	1.25	0.63
B12		Fr CH_2_Cl_2_	0.31	>5.000	5	5	1.25
B13	Te M L H_2_O	Fr H_2_O	2.5	1.25	0.31	0.31	0.16
B14		Fr CH_2_Cl_2_	2.5	0.63	0.16	0.08	0.08
B15	Te M L H_2_O/EthOH	Fr H_2_O	2.5	1.25	0.31	0.16	0.04
B16		Fr CH_2_Cl_2_	2.5	0.16	0.31	0.16	0.08
B17	Te M L EthOH	Fr H_2_O	2.5	0.63	0.63	0.31	0.31
B18		Fr CH_2_Cl_2_	0.63	0.31	0.08	0.04	0.04
B19	Te M Sb H_2_O	Fr H_2_O	1.25	0.63	0.16	0.08	0.08
B20		Fr CH_2_Cl_2_	0.63	0.31	0.16	0.16	0.08
B21	Te M Sb H_2_O/EthOH	Fr H_2_O	0.31	1.25	0.31	0.31	0.16
B22		Fr CH_2_Cl_2_	0.63	1.25	0.63	0.31	0.31
B23	Te M Sb EthOH	Fr H_2_O	0.63	1.25	0.31	0.16	0.16
B24		Fr CH_2_Cl_2_	1.25	1.25	0.31	0.16	0.16
B25	Mo T L H_2_O	Fr H_2_O	5	5	0.08	>5.000	>5.000
B26		Fr CH_2_Cl_2_	2.5	2.5	0.63	2.5	2.5
B27	Mo T L H_2_O/EthOH	Fr H_2_O	2.5	5	5	5	2.5
B28		Fr CH_2_Cl_2_	2.5	2.5	1.25	0.63	0.31
B29	Mo T L EthOH	Fr H_2_O	2.5	>5.000	2.5	5	5
B30		Fr CH_2_Cl_2_	2.5	2.5	0.63	0.63	0.63
**Codes**	**Sub-fractions**	**^a^ MIC (mg/mL)**					
		**Reference Strain**		**Isolates**			
		***C. albicans* NR-29450**		***C. albicans***	***C. glabrata***	***C. parapsilosis***	***Cr. neoformans***
C2	Te C L H_2_O/EthOH Fr H_2_O	sFr CH_3_OH	0.31	0.62	0.31	0.31	0.16
C4	Te C L H_2_O/EthOH Fr CH2Cl_2_	sFr CH_3_OH	0.31	0.62	0.31	0.62	0.31
C5	Te C L EthOH Fr CH_2_Cl_2_	sFr C_6_H_12_	0.62	1.25	0.62	1.25	0.62
C6		sFr CH_3_OH	1.25	1.25	1.25	>1.25	1.25
C8	Te C Sb H_2_O/EthOH Fr CH_2_Cl_2_	sFr CH_3_OH	0.16	1.25	0.31	0.62	0.08
C10	Te M L H_2_O Fr H_2_O	sFr CH_3_OH	0.16	0.62	0.31	0.31	0.16
C12	Te M L H_2_O Fr CH_2_Cl_2_	sFr CH_3_OH	0.31	0.62	0.08	0.08	0.08
C14	Te M L H_2_O/EthOH Fr H_2_O	sFr CH_3_OH	0.31	0.62	0.16	0.16	0.16
C15	Te M L H_2_O/EthOH FrCH_2_Cl_2_	sFr C_6_H_12_	0.31	0.31	0.31	0.31	0.16
C16		sFr CH_3_OH	0.16	0.16	0.16	0.62	0.16
C18	Te M L EthOH Fr H_2_O	sFr CH_3_OH	0.31	0.16	0.31	0.31	0.62
C20	Te M L EthOH Fr CH_2_Cl_2_	sFr CH_3_OH	0.16	0.16	0.31	0.31	0.31
C22	Te M Sb H_2_O Fr H_2_O	sFr CH_3_OH	0.16	0.08	0.08	0.16	0.04
C24	Te M Sb H_2_O Fr CH_2_Cl_2_	sFr CH_3_OH	0.16	0.08	0.08	0.16	0.04
C26	Te M Sb H_2_O/EthOH Fr H_2_O	sFr CH_3_OH	0.04	0.08	0.08	0.16	0.08
C28	Te M Sb H_2_O/EthOH FrCH_2_Cl_2_	sFr CH_3_OH	0.31	1.25	1.25	1.25	0.62
C30	Te M Sb EthOH Fr H_2_O	sFr CH_3_OH	0.31	0.31	0.31	0.31	0.31
C32	Te M Sb EthOH Fr CH_2_Cl_2_	sFr CH_3_OH	0.16	0.08	0.08	0.16	0.08
C33	Mo T L H_2_O/EthOH Fr CH_2_Cl_2_	sFr C_6_H_12_	>1.25	>1.25	>1.25	>1.25	>1.25
C34		sFr CH_3_OH	1.25	1.25	1.25	1.25	>1.25
C35	Mo T L EthOH Fr CH_2_Cl_2_	sFr C_6_H_12_	1.25	>1.25	>1.25	>1.25	1.25
C36		sFr CH_3_OH	1.25	0.62	0.62	>1.25	1.25
	Fluconazole (µg/mL)		0.50	1.00	8.00	8.00	2.00

^a^ Minimal Inhibitory Concentration (lowest concentration at which there is no visible growth of yeast); Mo T: *Monodora tenuifolia*; Te C: *Terminalia catappa*; Te M: *Terminalia mantaly*; L: leaves; Sb: stem bark; Tw: twigs; Br: branches; Pu: pulp; Se: seeds; PSe: seeds pericarp; FrPe: fruit pericarp; H_2_O: distilled water; H_2_O/EthOH: hydroethanol; EthOH: ethanol; Fr H_2_O: aqueous fraction; Fr CH_2_Cl_2_: methylene chloride fraction; sFr C_6_H_12_: Hexanic sub-fraction; sFr CH_3_OH: Methanolic sub-fraction. A1–A23: crude extracts; B1-B30: fractions; C2–C36: sub-fractions.

### 3.2. Activity of Bio-Guided Fractions

The first partition of the 15 selected extracts generated 30 fractions out of which 18 were progressed based on their MIC values (median MIC ≤ 0.62 mg/mL), including B3–B6, B10, B13–B24, and B28. Subsequent to the partition of the 18 fractions, seven sub-fractions (C2, C12, C22, C24, C26, C32 and C36) were afforded with median MIC ≤ 0.31 mg/mL. The most active sub-fraction (C26) showed a median MIC of 0.08 mg/mL. MICs of fractions and sub-fractions are summarized in Table 2.

Globally, there was a significant increase in activity from crude extracts to sub-fractions, with median MICs = 0.31 mg/mL for crude extracts; median MICs = 0.31 mg/mL for fractions; and median MICs = 0.08 mg/mL for sub-fractions. Extracts from *T. mantaly* showed more potency, while those from *M. tenuifolia* were the least active against the tested yeasts. There was no correlation between observed activities and solvents used for extraction.

**Table 2:** Effect of combined sub-fractions on the tested yeasts.

Combinations of sub-fractions	*C. albicans* NR-29450		*C. albicans*		*C. glabrata*		*C. parapsilosis*		*Cr. neoformans*	
	FICI	Int	FICI	Int	FICI	Int	FICI	Int	FICI	Int
C2/C12	1.06	I	1.06	I	1.06	I	1.00	A	0.62	A
C2/C22	0.75	A	0.50	S	0.75	A	0.62	A	0.31	S
C2/C24	0.56	A	0.75	A	0.75	A	0.53	A	0.31	S
C2/C26	0.62	A	0.75	A	0.75	A	1.12	I	0.62	A
C2/C32	0.56	A	1.12	I	0.75	A	1.00	A	0.37	S
C36/C2	0.50	A	0.62	A	0.50	S	0.50	S	0.50	S
C36/C12	0.37	S	0.62	A	0.56	A	0.37	S	0.25	S
C36/C22	0.53	A	0.62	A	0.75	A	0.75	A	0.37	S
C36/C24	1.00	S	1.12	I	0.50	S	0.75	A	0.50	S
C36/C26	0.75	A	0.75	A	0.62	A	0.56	A	0.25	S
C36/C32	0.62	S	0.50	S	0.75	A	0.56	A	0.62	A

FICI: Fractional Inhibitory Concentration Index; Int: interaction; I: indifference; A: additivity; S: synergy; C2: Te CL H_2_O/EthOH Fr H_2_O sFr CH_3_OH; C12: Te ML H_2_O Fr CH_2_Cl_2_ sFr CH_3_OH; C22: Te MSb H_2_O Fr H_2_O sFr CH_3_OH; C24: Te MSb H_2_O Fr CH_2_Cl_2_ sFr CH_3_OH; C26: Te MSbH_2_O/EthOH Fr H_2_O sFr CH_3_OH; C32: Te MSb EthOH Fr CH_2_Cl_2_ sFr CH_3_OH; C36: Mo TL EthOH Fr CH_2_Cl_2_ sFr CH_3_OH.

### 3.3. Activity of Combined Sub-Fractions

Eleven combinations were prepared from seven selected sub-fractions (C2, C12, C22, C24, C26, C32 and C36) and tested for their bioactivity. The seven sub-fractions used consisted of five from *T. mantaly*, one from *T. catappa* and one from *M. tenuifolia.* All the calculated fractional inhibitory concentrations (FICIs) showed values between 0.25 and 1.12, presenting synergy, additivity or indifference (Table 2). No case of antagonism was observed. Two combinations (C36/C2 and C36/C12) exerted above 50% synergistic interaction associated with more than 75% of MIC reduction. These combinations were prepared from a *M. tenuifolia* sub-fraction (C36) associated with each sub-fraction of *T. mantaly* (C12) and *T. catappa* (C2), respectively. They were therefore submitted to time kill kinetic assays.

### 3.4. Time Kill Kinetics of the Promising Sub-Fractions and Their Combinations

**Figure 1:**
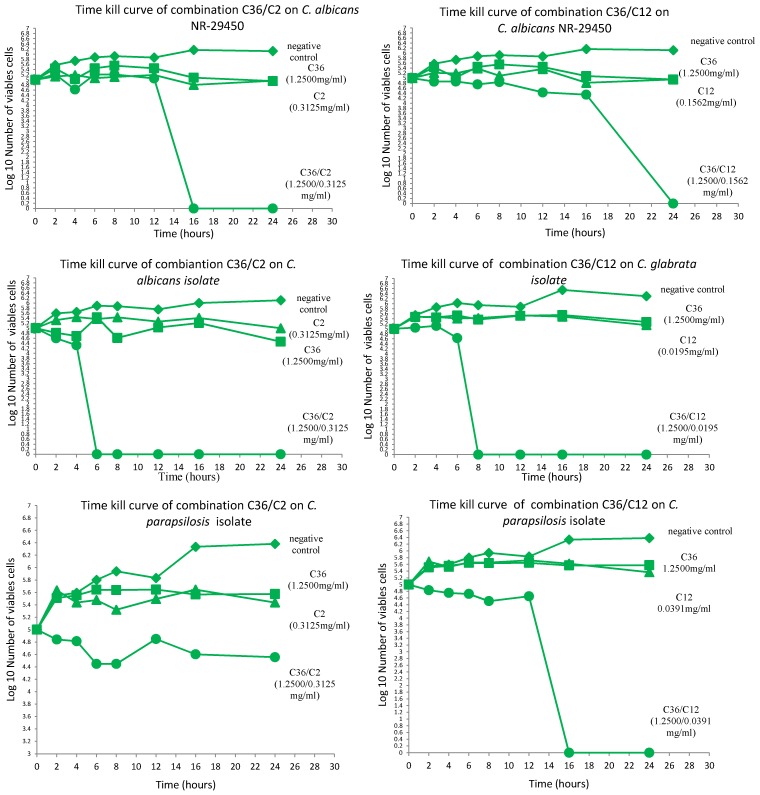

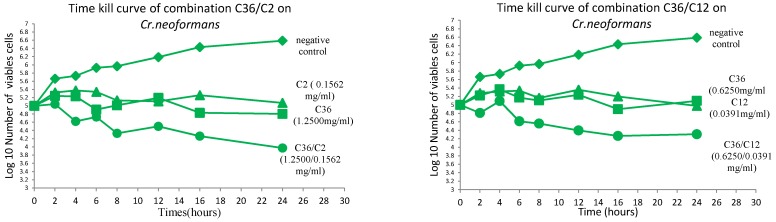
Time kill kinetic curves of combinations C36/C2 and C36/C12 against the tested yeasts. Sub-fractions were assessed alone and in combination for bioactivity at different time intervals.

The results obtained from the time kill assays indicated globally that all sub-fractions tested alone were fungistatic against all the microorganisms. On the contrary, the combinations exhibited fungicidal activity in most cases against the tested yeasts (Figure 1). Particularly, *C. albicans* and *C. glabrata* isolates were the most susceptible to the combinations C36/C2 and C36/C12, with all cells killed within 6 and 8 h of exposure, respectively. Comparatively, *C. albicans* NR-29450 was less sensitive to the combinations than the isolate. Overall, a fungicidal effect was obtained against the other microorganisms beyond 16 h. Only C36/C12 showed fungicidal action against *C. parapsilosis,* and the two combinations were fungistatic on *Cr. neoformans*.

### 3.5. Phytochemical Screening of Crude Extracts and Selected Sub-Fractions

The qualitative phytochemical screening of crude extracts and sub-fractions of interest showed the presence of alkaloids, flavonoids, tannins, saponins and steroids. However only two types of secondary metabolites (glucosides and steroids) were identified in all three most active sub-fractions (C2, C12, and C36), suggesting their implication in the exerted anti-yeast activity.

## 4. Discussion

The preparations of medicines by traditional healers are mainly carried out by boiling different organs of plants in water or macerating in alcoholic white wine. This information oriented the choice for extraction solvents. The antifungal activities obtained for *T. catappa*, *T. mantaly* and *M. tenuifolia* confirmed the traditional use of these plants in the treatment of fungal infections and also corroborated what had been previously found [39,40,41]. Aqueous and ethanolic extracts of stem bark of *T. catappa* and *T. mantaly* growing in the Cote d’Ivoire were shown to have anticandidal activity on clinical isolates of *C. albicans* with IC_50_ ranging from 0.02 to 0.55 mg/mL [39,40]. Furthermore, a comparative study showed that extracts from *T. mantaly* were more active than those from *T. catappa* against *Aspergillus fumigatus* [40]. To the best of our knowledge, this is the first scientific report on the antifungal potential of *M. tenuifolia* extracts. Also, the present work is pioneering on the aspect of optimizing activities by coupling bio-guided fractionation and extracts combinations. Based on modern trends in combining drugs for activity optimization [34,42,43], combinations of *T. mantaly* and *T. catappa* with *M. tenuifolia* sub-fractions have considerably increased the anti-yeast activity. This optimized action of the combinations is more likely due to components of the sub-fractions acting synergistically against the pathogens [33,44,45,46]. Moreover, the simultaneous actions of metabolites on different targets enhance their bioactivity and might also reduce the advent of resistance by the fungi. Results from the time kill kinetics assays showed that combinations increased the antifungal activity and also changed the pharmacokinetics of extracts from fungistatic to fungicidal. This finding is particularly significant, given that among the drawbacks of azole drugs, they are rather fungistatic. This profile probably contributed to the development of resistance of clinical isolates from immunocompromised patients to azoles, given that residual yeast cells are not cleared, enabling a positive selection of drug-resistant mutants [47]. The qualitative phytochemical screening of the promising sub-fractions (C2, C12 and C36) led to the identification of steroids and glucosides as common classes of secondary metabolites. Steroids have been reported to have antibacterial and antifungal properties. The correlation between membrane lipids and sensitivity for steroidal compounds indicates the mechanism through which steroids specifically associate with membrane lipids and exert their action by causing leakages from liposomes [48]. Apart from this, steroidal glucosides were shown to possess potent antifungal activity [49]. Needed antifungal drugs should be ideally fungicidal. To achieve this, promising combinations identified within the framework of this study should be rationally studied in detail and potentially developed into novel drugs to control yeast infections.

## 5. Conclusions

The results achieved from this work indicate that *T. mantaly*, *T. catappa* and *M. tenuifolia* are potential sources of new antifungal drugs. The results also show improved potency in correlation with fractionation of extracts, as well as the combination of sub-fractions. The more promising combinations exerted synergistic and fungicidal actions against the tested yeasts. Overall, these findings support the continued investigation of extracts from the studied plants using the bio-guided fractionation and combination strategies to develop innovative therapies against mycoses.
